# TRIM8 as a predictor for prognosis in childhood acute lymphoblastic leukemia based on a signature of neutrophil extracellular traps

**DOI:** 10.3389/fonc.2024.1427776

**Published:** 2024-08-19

**Authors:** Waihin Tin, Cuilan Xiao, Kexin Sun, Yijun Zhao, Mengyun Xie, Jiayin Zheng, Ying Wang, Sixi Liu, Uet Yu

**Affiliations:** ^1^ Department of Pediatrics, The First Affiliated Hospital, Sun Yat-sen University, Guangzhou, Guangdong, China; ^2^ Department of Oncology, The Second Clinical College of Guangzhou University of Chinese Medicine, Guangzhou, China; ^3^ Department of Maternal and Child Health of Haizhu District, Guangzhou, China; ^4^ Key Laboratory of Stem Cells and Tissue Engineering (Ministry of Education), Zhongshan School of Medicine, Sun Yat-sen University, Guangzhou, Guangdong, China; ^5^ Sun Yat-sen Memorial Hospital, Sun Yat-sen University, Guangzhou, Guangdong, China; ^6^ Department of Hematology, Guangdong Pharmaceutical University, Guangzhou, Guangdong, China; ^7^ Department of Hematology and Oncology, Shenzhen Children’s Hospital, Shenzhen, China

**Keywords:** acute lymphoblastic leukemia, neutrophil extracellular traps, prognostic model, NET-related genes, TRIM8

## Abstract

**Background:**

Neutrophil extracellular traps (NETs) can be attributed to the metastasis, occurrence, and immune evasion of cancer cells. We investigated the prognostic value of NET-related genes in childhood acute lymphoblastic leukemia (cALL) patients.

**Methods:**

Differential gene expression analysis was conducted on samples collected from public databases. Grouping them based on the expression level of NET-related genes, we assessed the correlation between immune cell types and the risk score for having a poor prognosis of cALL, with an evaluation of the sensitivity of drugs used in cALL. We further divided the groups, integrating survival data. Subsequently, methods including multivariable Cox algorithms, least absolute shrinkage and selection operator (LASSO), and univariable were utilized to create a risk model predicting prognosis. Experiments in cell lines and animals were performed to explore the functions of TRIM8, a gene selected by the model. To validate the role of TRIM8 in leukemia development, lentivirus-mediated overexpression or knockdown of TRIM8 was employed in mice with T-ALL and B-ALL.

**Results:**

Kaplan–Meier (KM) analysis underscored the importance of differentially expressed genes identified in the groups divided by genes participated in NETs, with enrichment analysis showing the mechanism. Correlation analysis revealed significant associations with B cells, NK cells, mast cells, T cells, plasma cells, dendritic cells, and monocytes. The IC_50_ values of drugs such as all-trans-retinoic acid (ATRA), axitinib, doxorubicin, methotrexate, sorafenib, and vinblastine were increased, while dasatinib exhibited a lower IC_50_. A total of 13 NET-related genes were selected in constructing the risk model. In the training, testing, and merged cohorts, KM analysis demonstrated significantly improved survival for low-risk cALL patients compared to high-risk cALL patients (*p* < 0.001). The area under the curve (AUC) indicated strong predictive performance. Experiments in Jurkat and SUP-B15 revealed that TRIM8 knockdown decreased the proliferation of leukemia cell lines. Further experiments demonstrated a more favorable prognosis in mice with TRIM8-knockdown leukemia cells. Results of cell lines and animals showed better outcomes in prognosis when TRIM8 was knocked down.

**Conclusion:**

We identified a novelty in a prognostic model that could aid in the development of personalized treatments for cALL patients. Furthermore, it revealed that the expression of TRIM8 is a contributing factor to the proliferation of leukemia cells and worsens the prognosis of cALL.

## 1 Introduction

Acute lymphoblastic leukemia (ALL) is a common cancer in children, as approximately 25% of cancers are diagnosed under the age of 15 (1, 2). Additionally, approximately half of all ALL cases occur in children and adolescents, making it the most common acute leukemia before the age of 20 (3). Childhood ALL (cALL) has been considered a successful example of treatment in pediatric oncology, with survival rates increasing from around 10% in the 1960s to 90% today (3). Although most children with ALL have a high survival rate under current treatment protocols, the prognosis for relapsed and refractory ALL remains low, making it the main cause of death for children. Therefore, there is an urgent need for new treatment targets and insights into relapsed and refractory cALL (4).

Neutrophil extracellular traps (NETs), regulated by the reactive oxygen species (ROS) mediated by nicotinamide adenine dinucleotide phosphate hydrogen (NADPH) oxidase and histone citrullination, are a defense mechanism in the immune system (5–7). Initial studies suggested that NETs functioned in capturing microbes and defending against microbial threats in the human body; however, further research has revealed the promoting effects of NETs on cancer, manifested by increased inflammatory factors and growth factors secreted by neutrophils in the tumor microenvironment (8). NET formation, reported in a previous study, can protect tumor cells from immune cell killing, such as CD8 T cells and NK cells, playing a crucial role in tumor metastasis (9). Previous reports have shown that NETs are attributed to hematopoietic malignancy, and a clinical study has revealed that NETs are activated in ALL, suggesting a potential involvement of NETs in the adverse prognosis of ALL (10–13). However, researchers have focused on the role of neutrophils in infection events during the treatment of ALL before, neglecting their potential effects on protecting the tumor cells.

Therefore, our study investigated the relationship between NETs and cALL, exploring its role in the tumor microenvironment, immune cell communication, and potential drug resistance (14, 15). To precisely predict the prognosis of cALL, we constructed a model for predicting the prognosis of cALL and performed *in vivo* and *in vitro* experiments to validate the gene in the prognostic model.

## 2 Materials and methods

### 2.1 Data resources

We obtained mRNA sequencing data from 668 patients with cALL by downloading it from “Therapeutically Applicable Research to Generate Effective Treatments (TARGET)”, a program within The Cancer Genome Atlas (TCGA) in this article. TARGET is a program using “multi-omic “ methods to understand the molecular profiles of childhood cancers. We collected a comprehensive set of clinical information for these patients, along with the expression levels of 67 genes known to be involved in NETs, which were selected based on previous studies. Detailed information on the patients and genes can be found in **Supplementary Table S1**. The clinical information of patients can be found in **Supplementary Table S2**.

### 2.2 Data processing

Normalization of the sequencing data from TCGA was processed with the R package “limma “. Next, the differentially expressed genes (DEGs) were screened out with | fold change of log_2_ | > 1 and adjusted *p* < 0.05 between two groups divided by the expression level of 67 genes (16).

### 2.3 Analysis for clustering

Using R packages, namely “ConsensusClusterPlus “ and “ggplot2 “, we performed grouping based on the expressed levels of 67 genes involved in NETs and DEGs combined with the hazard ratio. The samples were classified, and then a principal component analysis (PCA) was conducted to evaluate the clustering (17).

### 2.4 Survival and enrichment analysis

Installing the “survival “ package in R software, we further investigated the significance of the consensus clustering in clinical value and conducted a Kaplan–Meier survival analysis to analyze the differences between clusters of NET-related genes. Additionally, we explored the correlations between the NET genes and prognosis. To gain further insights into the biological implications of the NET genes, we downloaded two gene sets, namely “c2.cp.kegg.v7.4.symbols.gmt “ and “immune.gmt “, from the Molecular Signatures Database (MSigDB). These gene sets were prepared for gene set variation analysis (GSVA) and single sample gene set enrichment analysis (ssGSEA), with the function “GSVA “ performing enrichment analysis based on gene sets of interest (18).

### 2.5 Drug sensitivity and correlation analysis

With document “Cibersort.R “ and R packages “preprocessCore “ and “parallel “, the correlation between the risk score and immune cell types was analyzed by Spearman’s rank correlation test. The sensitivity data of drugs was offered by the “pRRophetic “ R package. The packages predict the sensitivity of drugs based on the data from cancer cell lines, calculating the IC_50_ for each drug (19).

### 2.6 Evaluation of prognostic model

Integrating the expression data of DEGs in cALL patients with their corresponding survival data, we randomly divided the samples into two groups after estimating the hazard ratio using Cox proportional hazards regression. The training group consisted of 285 patients. In the training group, univariate Cox regression analysis (*p* < 0.05) on NET-related genes was performed to identify potential candidate genes. The least absolute shrinkage and selection operator (LASSO) Cox regression then adjusted the overfitting and selected the important genes in the model. Following multivariable Cox regression, we calculated the coefficients for the prognostic model and examined the cruciality of each clinical character. In addition, we divided the cALL patients from the training group into two groups, namely, the low-risk group and the high-risk group, and the criteria for grouping depended on the median risk score calculated. The effectiveness of the prognostic risk model was evaluated using the following methods: Kaplan–Meier analysis, receiver operating characteristic (ROC) curve, distribution of risk scores, heatmap of genes, and survival status. In analyzing gene ontology (GO) and the Kyoto Encyclopedia of Genes and Genomes (KEGG), the R package “clusterProfiler “ was performed on the screened genes. These results provided insights into the functional annotations and pathways associated with the identified genes.

### 2.7 Cell lines and culture conditions

The media for SUP-B15, Jurkat, and 293T cells were Iscove’s modified Dulbecco’s medium (IMDM; Sigma-Aldrich, China; Merck KGaA, China) with 20% fetal bovine serum (FBS; Gibco, China; Thermo Fisher Scientific Inc., China), RPMI-1640 medium (1640; Sigma-Aldrich, China; Merck KGaA, China) supplemented with 10% FBS, and Dulbecco’s modified Eagle’s medium (DMEM; Sigma-Aldrich; Merck KGaA) with 10% FBS, respectively (20). Cell lines were maintained in a medium containing 1% penicillin–streptomycin (Gibco). To knock down the expression of TRIM8, a specific short-hairpin RNA (shRNA) sequence was used. The shRNA sequence for TRIM8 was as follows: Forward: CCGG CCAGTACTGCTGCTACTACAG CTCGAG CTGTAGTAGCAGCAGTACTGG TTTTG; Reverse: AATT CAAAACCAGTACTGCTGCTACTACAG CTCGAG CTGTAGTAGCAGCAGTACTGG. The scrambled shRNA sequence was referred to as this plasmid (Plasmid No. 1864). The restriction endonucleases *Eco*RI and *Age*I were used for cloning. The TRIM8 knockdown in SUP-B15 cells was achieved using lentiviral transduction, with psPAX2 (Plasmid No. 12260), pMD2.G (Plasmid No. 12259), and pLKO.1-TRC control (Plasmid No. 10879) containing “EGFP “ and “PUROR “. With 8 µg/ml of polybrene (Sigma-Aldrich; Merck KGaA), the mixture of cells and polybrene was transduced by centrifugation (1,000 rpm) at 37°C for 1.5 h. After selecting the cells with 1 µg/ml puromycin (ST551, Beyotime, China) for 48 h, we validated the knockdown efficiency of TRIM8 by using qPCR.

### 2.8 qPCR and ROS assay

The RNA isolation and ROS assay were performed according to the manufacturer’s instructions (Q221-01, RC102-01, Vazyme, China; S0033S, Beyotime, China). The process of qPCR was performed on the Bio-Rad CFX96 TouchTM Real-Time PCR Detection system. The analysis of ROS was completed using Attune NxT (Thermo Fisher).

### 2.9 Mice and ALL model construction

All mice used in the experiments were between 8 weeks and 12 weeks old, with a C57BL/6J genetic background. The models of T-ALL and B-ALL were constructed by different viruses. In T-ALL, a plasmid containing the sequencing of MSCV-NOTCH1-IRES-GFP-TAA was packaged by the virus packaging plasmid Ecopack. Two plasmids were mixed with polyethylenimine (23966, Polysciences, China) and then added to 293T cells. The medium was removed after 6 h, and the supernatant was collected at 48 h and 72 h. Bone marrow (BM) cells were collected from mice preinjected with 5-FU (51-21-8, Sigma-Aldrich; Merck) once to enrich the hematopoietic stem and progenitor cells. The cells were collected from the bone marrow at day 5. 5 in preparation for transduction using previously packaged virus. Infected BM cells were infected with retroviral medium containing 8  μg/ml polybrene (40804ES86, Yeasen, China). The infection was repeated after 24 h to generate NOTCH1-infected preleukemia cells. The leukemia cells (4 × 10^5^) were then transplanted into lethally irradiated (9 Gy) mice by intravenous injection. For the B-ALL model, the key sequencing was replaced from NOTCH1 to N- MYC, and the following steps were in line with the modeling of T-ALL mice. The vector of lentiviruses expressing shRNAs for TRIM8 was mentioned before, with packaging plasmid psPAX2 and pMD2.G. The transfection process was previously described. The plasmids were gifts from the laboratory of MengZhao (21).

### 2.10 Transplantation of leukemia cells

For the infection of primary leukemia cells, a mixture of medium containing the virus and leukemia cells (2 × 10^6^) was plated in 12-well dishes with RPMI medium, 10% FBS, and cytokines as follows: SCF (10 ng/ml), IL-3 (10 ng/ml), IL-6 (10 ng/ml), and GM-CSF (10 ng/ml). After centrifugation for 1.5 h (768×*g*) (22), the medium was removed 2 h later. Next, the cells were selected with 2 μg/ml of puromycin after 2 days. The leukemia cells used in the last step were the BM cells collected from previously established leukemia mice. Subsequently, the cells were injected into the tail vein of sublethal irradiated mice (4.5 Gy). The data, including the survival rate and weight of the recipient mice, were recorded during the experiment. The data on leukemic cells in BM, peripheral blood (PB), and spleen were recorded using flow cytometry. All animal experiments performed in this research were conducted with the approved protocols by the Institutional Animal Care and Use Committee (agreement number: SYSU-IACUC-2024-B0726).

## 3 Results

### 3.1 Identification of differentially expressed genes in childhood acute lymphoblastic leukemia based on the signature of neutrophil extracellular traps

**Figure 1:**
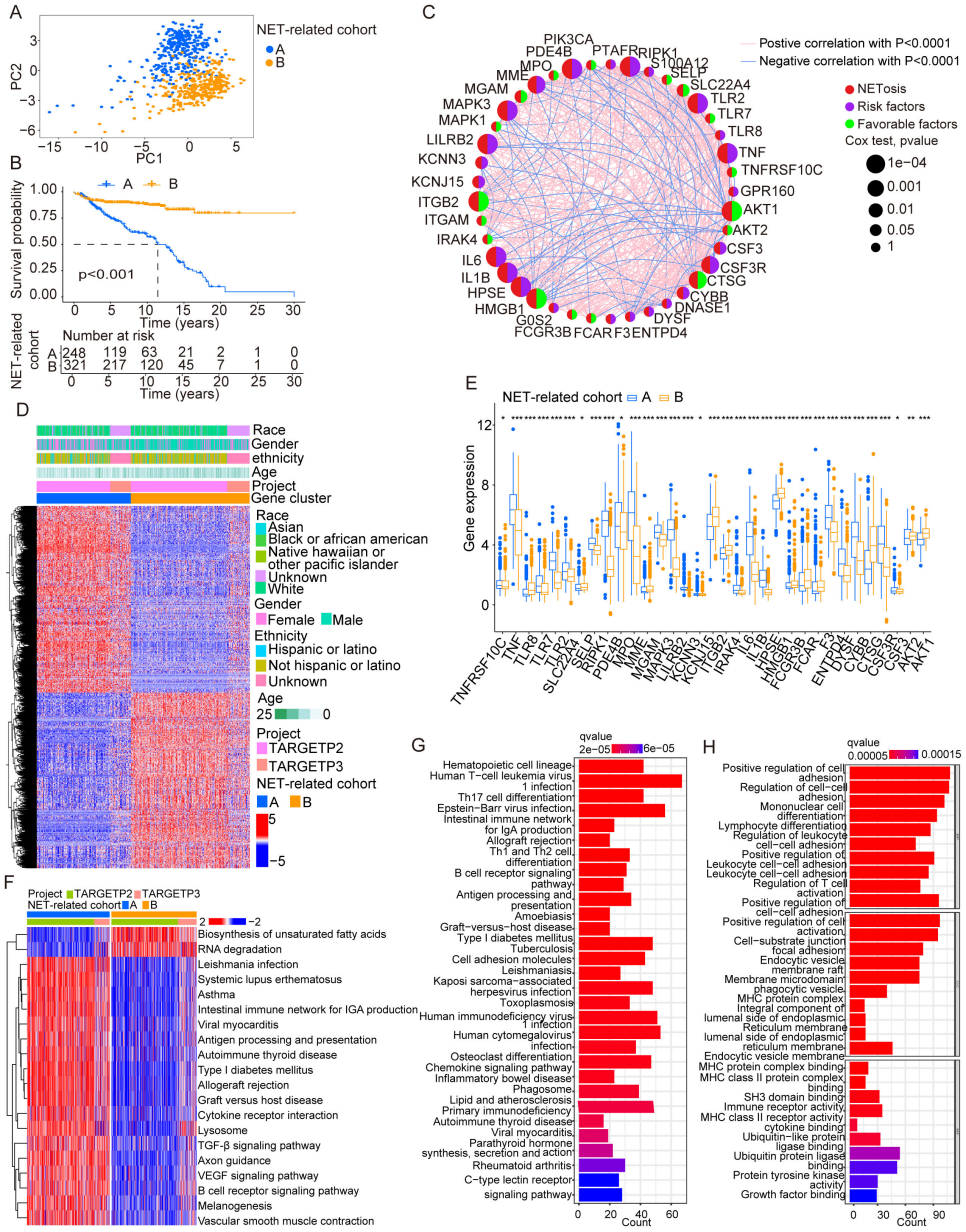
Identification and traits of NET-related genes in cALL. **(A)** The PCA plot showing the distribution between groups divided by NET-related genes. **(B)** KM cures in two groups separated by NET-related genes. **(C)** Network plot showing NET-related genes in cALL. **(D)** Heatmap showing the distribution of DEGs of NET-related genes in cALL. **(E)** Box plot showing the significant genes between cohorts of NET-related genes. ^*^ *p* < 0.05; ^**^ *p* < 0.01; ^***^ *p* < 0.001. **(F)** Plot of GSVA performed on cohorts of NET-related genes. **(G, H)** Analysis of GSEA in cohorts of NET-related genes.

We integrated 67 genes that participated in NETs according to studies reported before and performed analyses using bioinformation integrated from the TCGA, exploring the prognostic potential of these genes. Samples were divided into two groups according to the expressed levels of 67 genes (**Supplementary Table S1**). We then confirmed the DEGs analyzed between two cohorts and further classified samples with NET-related genes (**Figure 1A**). The figure illustrates the significant difference in KM curves between the two groups (**Figure 1B**), and the following networks showed the correlation in NET-related genes (**Figure 1C**). The following heatmap showed the distribution among NET-related genes in patients with cALL (**Figure 1D**). The following figure shows the significant difference in genes that participated in NETs between the two groups (**Figure 1E**). Interestingly, analysis of pathways using GSVA indicated that NET-related genes play a crucial role in the function of the immune system (**Figure 1F**). The enrichments in the communication of immune cells from the KEGG and GO analyses reveal the potential of NET-related genes in the differentiation of hematopoietic cells and immune cells (**Figures 1G, H**).

### 3.2 Analysis of immune cells and drug sensitivity

**Figure 2:**
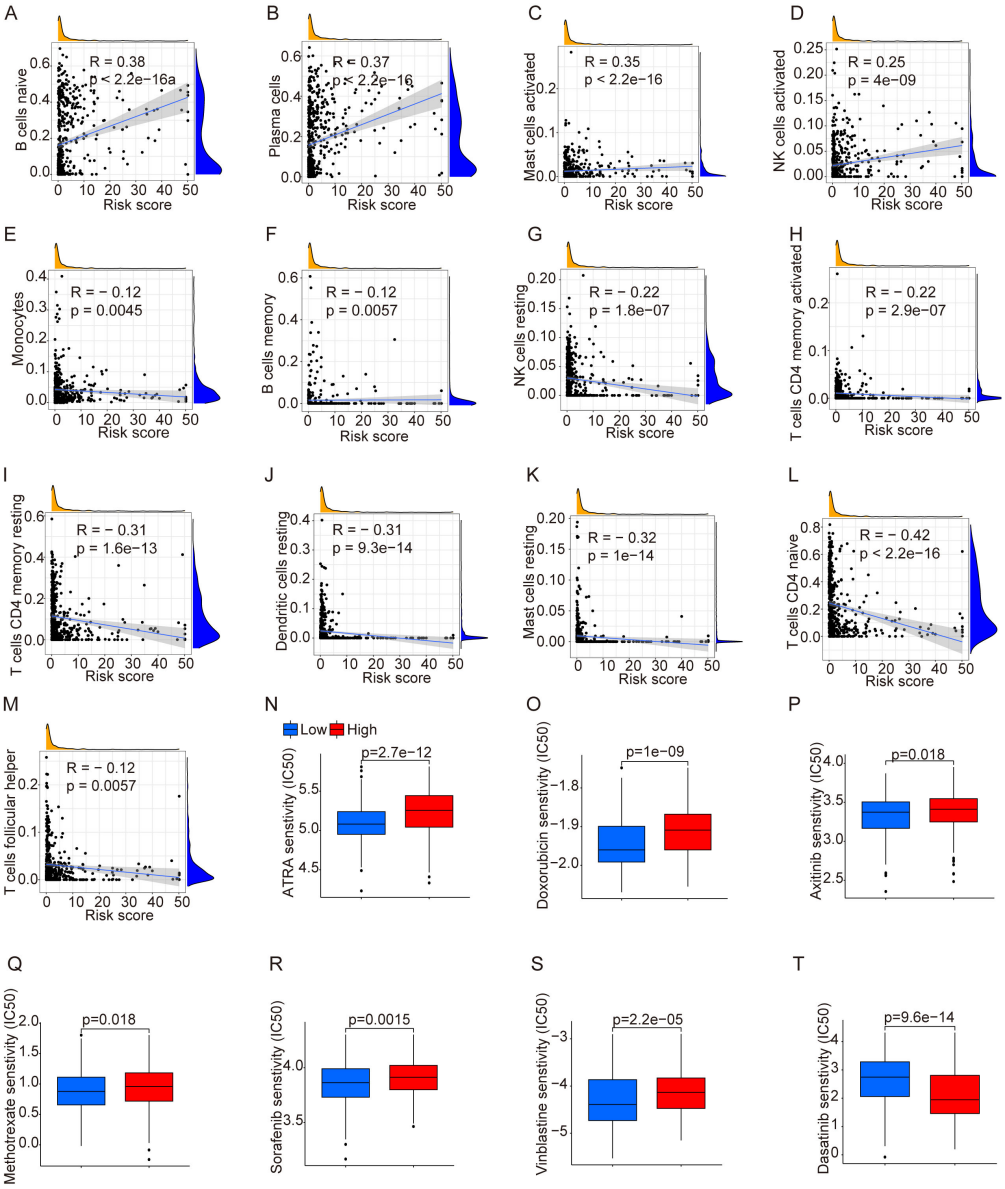
Analysis of immune cells and drug sensitivity. **(A–M)** Correlation between immune cells and risk score. **(N–T)** Drug sensitivity between groups divided by risks.

In previous figures, NET-related genes have been found to be associated with significant differences in prognosis. As previous results suggest their involvement in cell communication, we conducted a correlation analysis between immune cells and risk scores. We found that cells including B cells (naive), plasma cells, mast cells (activated), and NK cells (activated) were positively correlated with risk scores, while monocytes, B cells (memory), NK cells (resting), CD4 T cells (memory activated), CD4 T cells (resting memory), dendritic cells (resting), mast cells (resting), CD4 T cells (naive), and T cells (follicular helper) were negatively correlated (**Figures 2A–M**). Additionally, due to chemotherapy drug resistance in relapsed cALL, we evaluated the potential sensitivity of drugs commonly used in the treatment of leukemia. Patients expressing high-risk genes showed resistance to drugs such as ATRA, doxorubicin, axitinib, methotrexate, sorafenib, and vinblastine. However, dasatinib showed a lower IC_50_, indicating its potential therapeutic effects in relapsed cALL (**Figure 2T**).

### 3.3 Evaluation for prognostic model based on genes related to neutrophil extracellular traps in patients with childhood acute lymphoblastic leukemia

**Figure 3:**
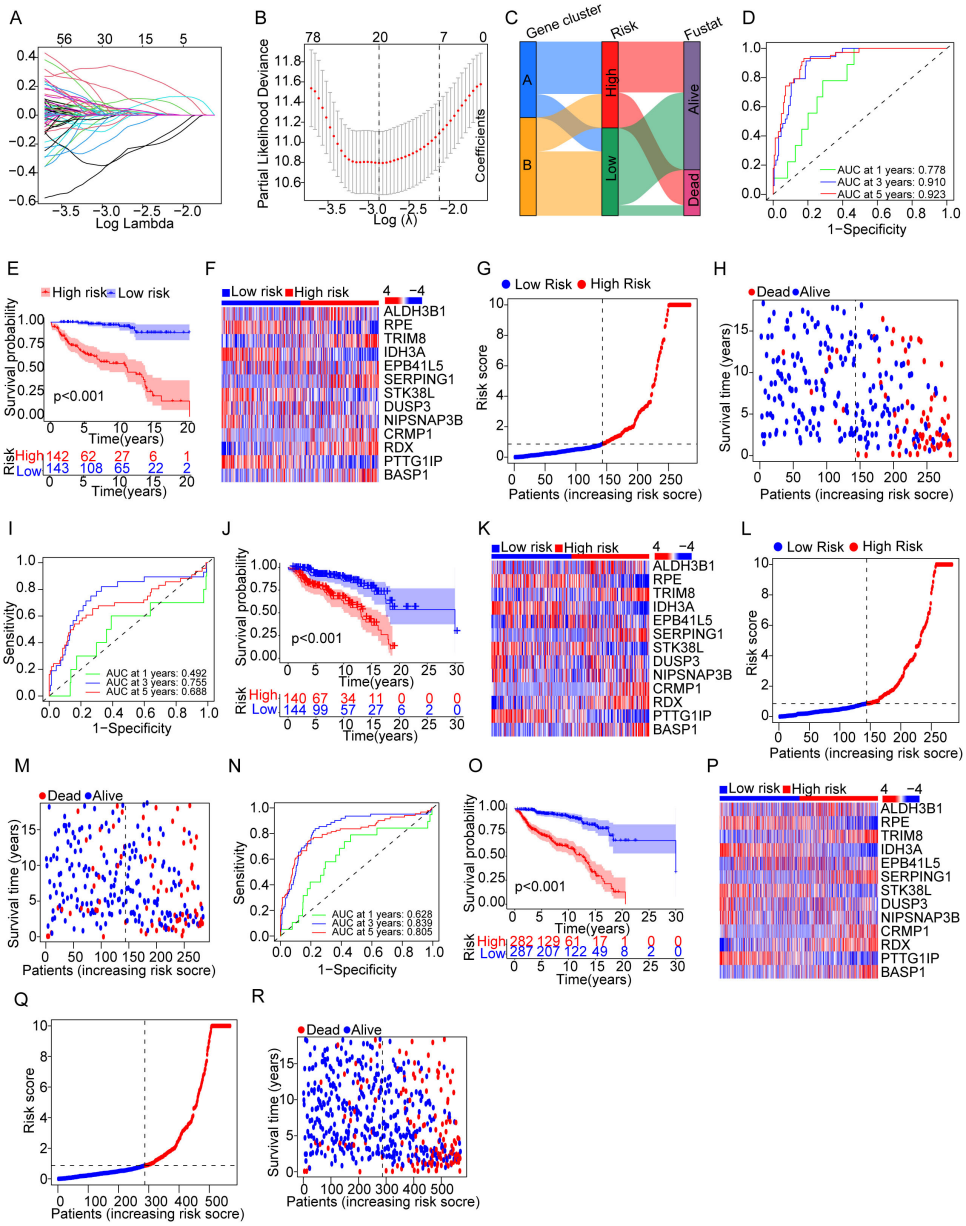
Evaluation for a prognostic model of genes related to NETs in patients with cALL. **(A)** Plots of LASSO selecting candidate genes. **(B)** Cross-validation for LASSO. **(C)** Alluvial diagram of distributions in different groups. **(D–G)** ROC, KM curve, heatmap related to selected NET-related genes, and plots showing distribution between risk score and patients; **(H)** plots showing distribution between survival status and patients in the training group. **(I–R)** The types of **(I–R)** are consistent with **(D–H)**, but the group is a testing group and a merging group, respectively.

The results mentioned before demonstrated the potential effects of the NET-related genes on the prognosis of cALL. To accurately assess the prognosis of cALL, a prognostic risk model was constructed. Data of samples were randomly divided into testing (*n* = 284) and training groups (*n* = 285). After using Cox regression algorithm and LASSO Cox regression algorithm, 13 genes were selected as the most powerful predictive factors (namely, TRIM8, ALDH3B1, RPE, IDH3A, EPB41L5, SERPING1, STK38L, DUSP3, NIPSNAP3, CRMP1, RDX, PTTG1IP, and BASP1), with the formula shown in **Supplementary Table S2**. The model was accomplished by considering the expression of genes, hazard ratio, and survival status (**Figures 3A–C**). We validated the model using multiple methods. The differences in overall survival (OS) among the three groups, namely, the training group, testing group, and merging group (combining training and testing groups), were significant, with *p* < 0.001 among the three groups. The AUCs of the ROC curves for the training group at 1 year, 3 years, and 5 years were 0.778, 0.910, and 0. 923, respectively, while the AUCs for the testing group were 0.492, 0.755, and 0. 688, respectively. Overall, the AUCs for the merging group were 0.628, 0.839, and 0. 805, respectively (**Figures 3D–R**).

### 3.4 Evaluating for nomogram prognostic model

**Figure 4:**
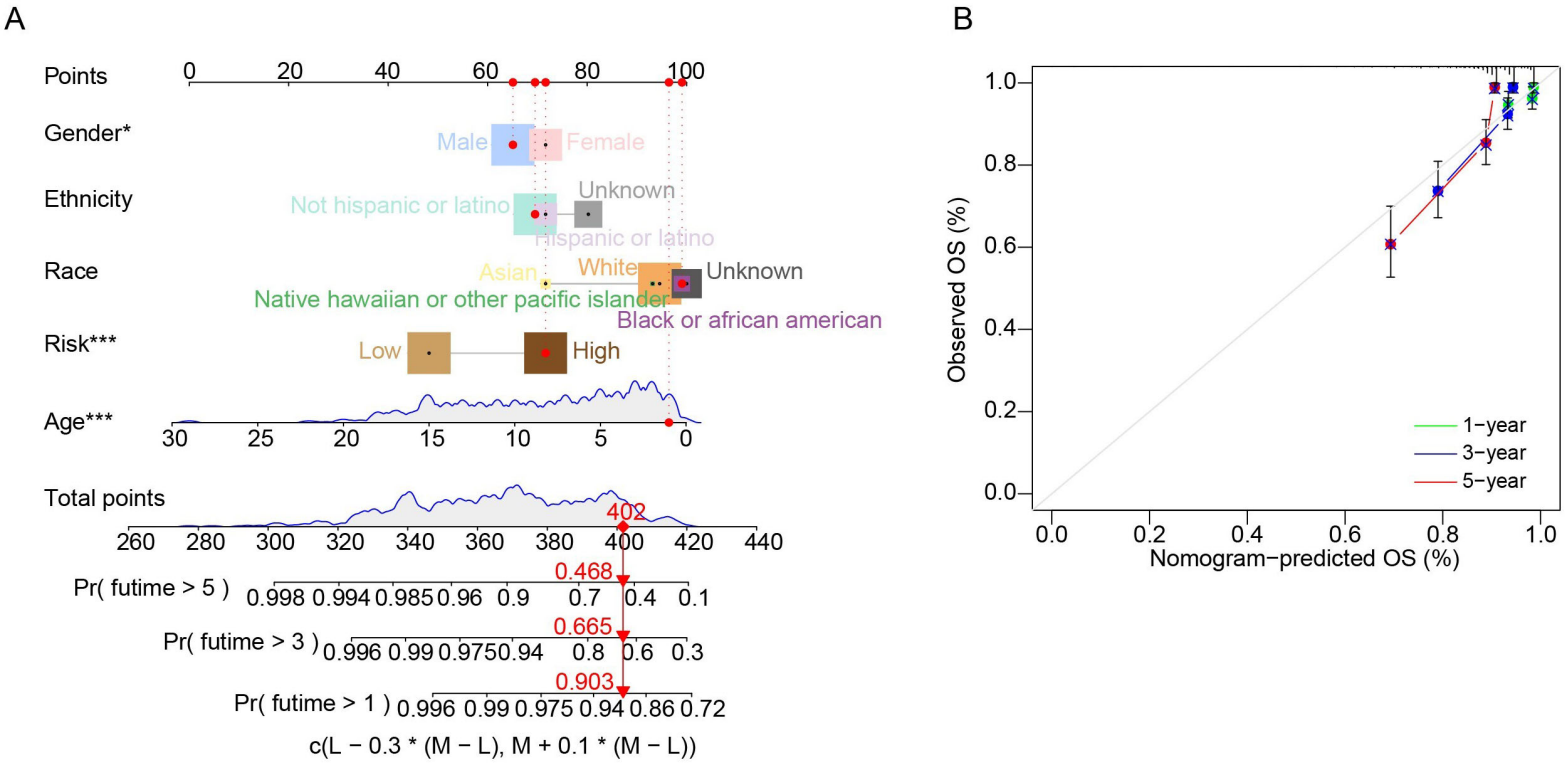
Evaluating for nomogram model. **(A)** Nomogram in predicting 1-year, 3-year, and 5- year OS in patients with cALL. **(B)** Calibration curves of the nomogram.

We performed univariate Cox regression analysis to investigate the prognostic value of the risk score by matching the risk score of cALL patients in the merging cohort (*n* = 569) with parameters recorded in the clinical data, such as gender, ethnicity, race, age, and risk. These five parameters were added to a nomogram, which demonstrated good predictive efficacy for personal prognostic outcomes at first, third, and fifth years (**Figure 4A**). Calibration curves were then constructed to assess the consistency of the model in predicting survival probabilities (**Figure 4B**).

### 3.5 Validation in cell lines and mice of childhood acute lymphoblastic leukemia

**Figure 5:**
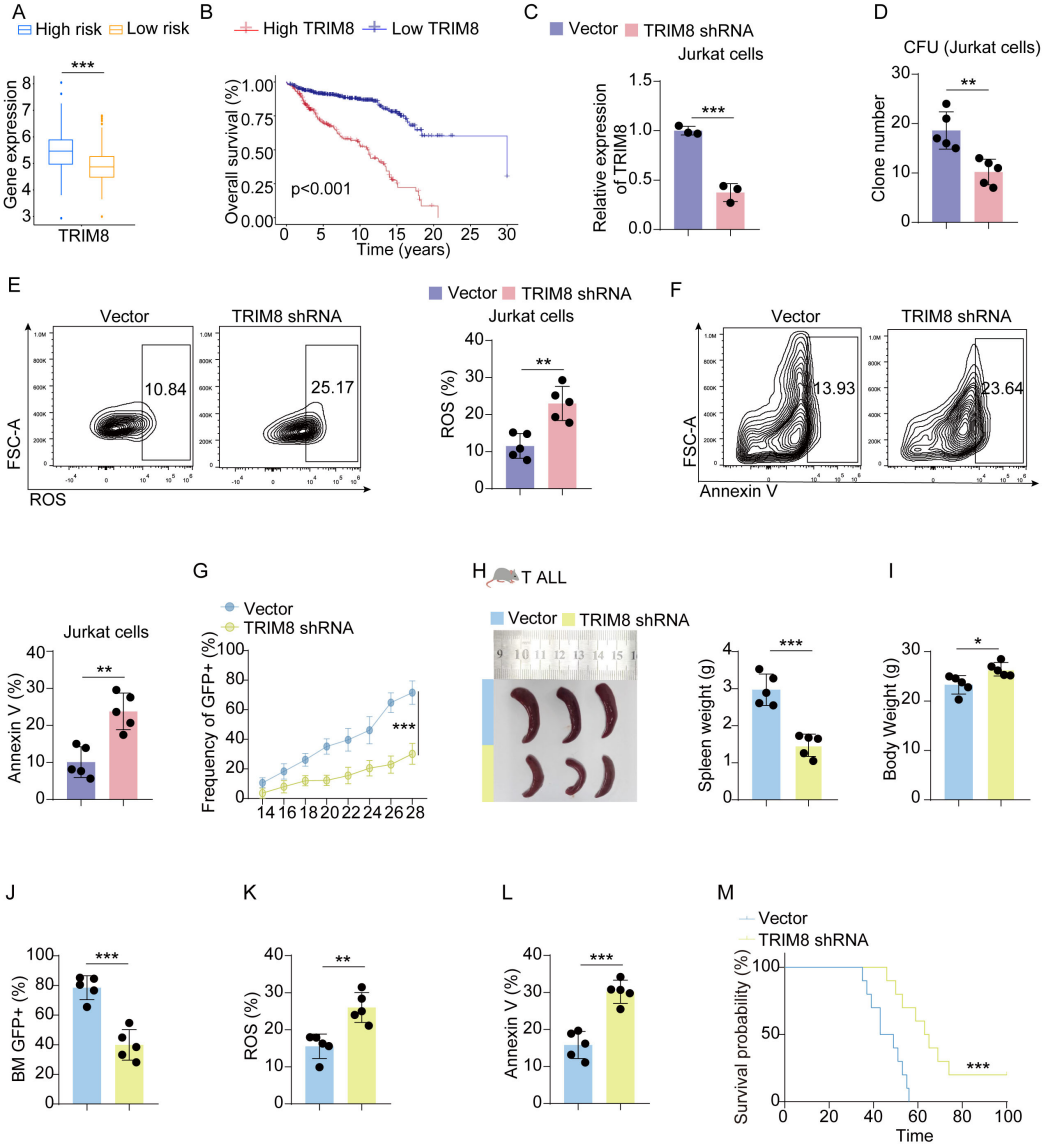
Validation in T-ALL cell lines and mice. **(A)** Box plot of the expression level of TRIM8 in high- and low-risk groups in Jurkat cells. **(B)** KM curves for groups divided by median expression levels of TRIM8. **(C)** Results of qPCR for TRIM8 knockdown Jurkat cells. **(D)** Colony-forming units for TRIM8 knockdown Jurkat cells. **(E, F)** Analysis of ROS level and apoptosis for TRIM8 knockdown Jurkat cells. **(G)** Percentages of leukemia cells with GFP taken in peripheral blood. **(H, I)** Results of spleen size, spleen weight, and body weight. **(J)** Percentages of leukemia cells with GFP taken in bone marrow. **(K**, **L)** The results of ROS and apoptosis for bone marrow cells from T-ALL mice. **(M)** Survival curves for T-ALL mice in groups of vector and TRIM8 knockdown. ^*^ *p* < 0.05; ^**^ *p* < 0.01; ^***^ *p* < 0.001.

**Figure 6:**
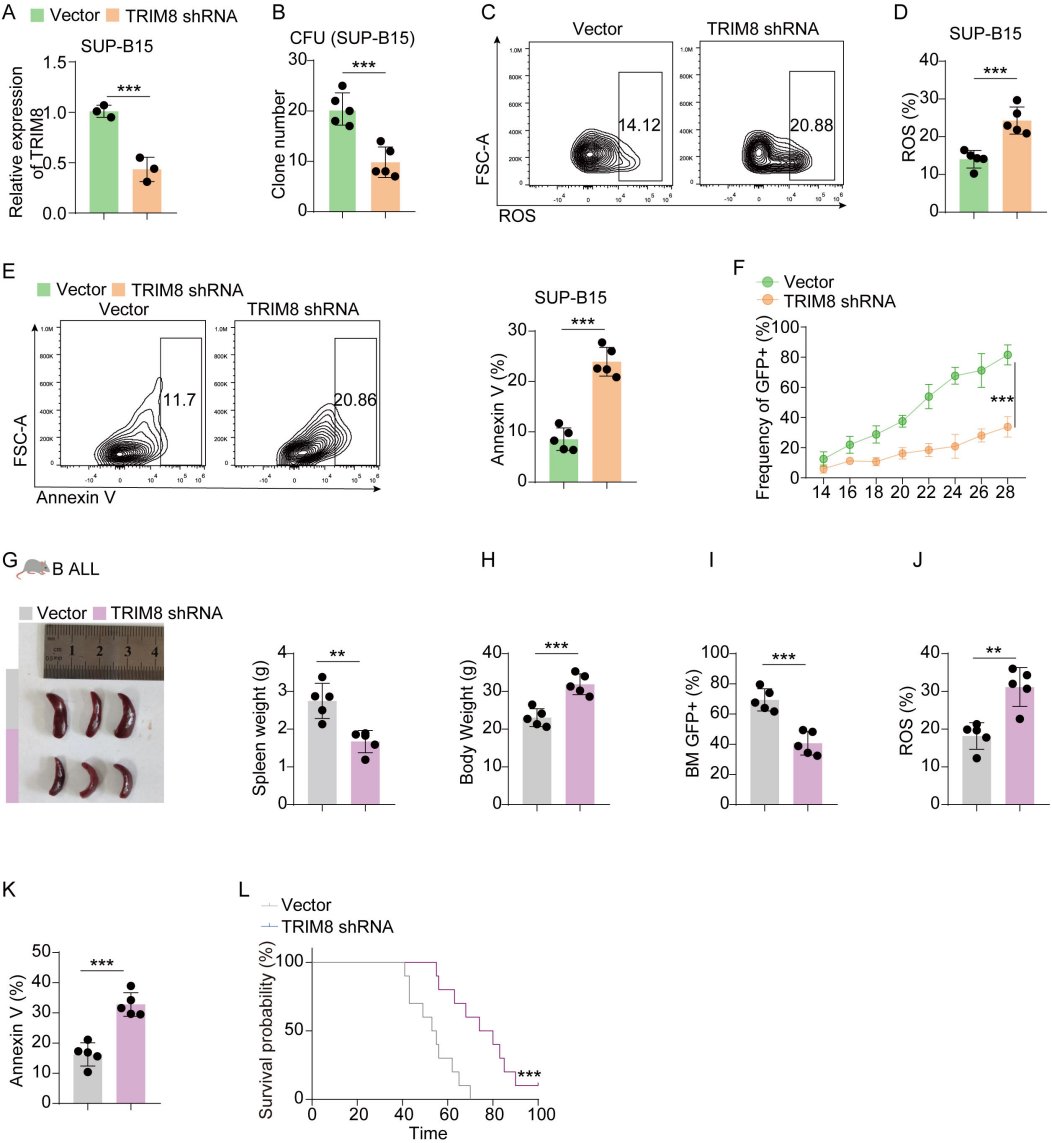
Validation in B-ALL cell lines and mice. **(A)** Results of qPCR for TRIM8 knockdown SUP-B15 cells. **(B)** Clone-forming units for TRIM8 knockdown SUP-B15 cells. **(C–E)** Analysis of ROS level and apoptosis for TRIM8 knockdown SUP-B15 cells. **(F)** Percentages of leukemia cells with GFP taken in peripheral blood. **(G, H)** Results of spleen size, spleen weight, and body weight. **(I)** Percentages of leukemia cells with GFP taken from bone marrow. **(J, K)** The results of ROS and apoptosis for bone marrow cells from B-ALL mice. **(L)** Survival curves for B-ALL mice in groups of vector and TRIM8 knockdown. ^**^ *p* < 0.01; ^***^ *p* < 0.001.

Before conducting experiments, we observed that the expression of TRIM8 was higher in the high-risk group, and the differential expression of TRIM8 contributed to the survival difference between the two groups (**Figures 5A, B**). Due to the significant prognostic value of TRIM8 and its weight in the formula, we performed experiments to validate its role in cALL. Initially, we constructed shRNA to knock down TRIM8 in Jurkat cell lines (**Figure 5C**). Compared to the control group, we observed a significant reduction in tumor cell proliferation, along with increased levels of ROS and apoptosis (**Figures 5D, E**). These results indicated the involvement of TRIM8 in tumor proliferation. To further investigate the role of TRIM8, we employed animal models. We established a T-ALL leukemia model in C57 mice with TRIM8 knockdown leukemia cells. The prognosis in T-ALL mice was consistent with the bioinformatics prediction. Mice with TRIM8 gene knockdown in leukemia cells exhibited a significant decrease in the percentage of leukemia cells in peripheral blood, a noticeable reduction in spleen size and weight, and an overall increase in body weight (**Figures 5F–H**). Examination of harvested bone marrow cells showed a significant decrease in leukemia cell count, and the results of the ROS and annexin V assays were consistent with the trends observed in the cell experiments. Similar trends were observed in the B-ALL cell line and mice (**Figure 6**).

## 4 Discussion

Previous studies have demonstrated the functions of NETs in cancer development and progression. Although NETs have been extensively studied in various cancer types (23–25), the role of NETs remains unclear in leukemia. Despite the significant advancements in the treatment for patients with cALL (3), there are patients who still do not benefit from existing therapies, showing deteriorating prognoses. Therefore, exploring the potential contribution of NETs in the recurrence of cALL holds promise for uncovering novel therapeutic insights.

In this research, we used multiple methods including analysis from bioinformation and experiments to investigate the role of NET-related genes in cALL and examine their expression patterns in relation to immune response, drug resistance, and prognosis. To establish a reliable prognostic model, we collected sequencing data from the TCGA databases and performed comprehensive analyses on groups of NET-related genes. Separating patients into high-risk and low-risk groups according to the expression level of genes, we observed significant differences in prognosis. The functional enrichment analysis of DEGs revealed their association with neutrophil-related functions, such as immune cell activation, cellular communication, and differentiation of hematopoietic cells. Interestingly, this enrichment indicated a potential link between NET-related genes and impaired hematopoietic cell differentiation, leading to abnormal proliferation of hematopoietic cells and the onset of leukemia.

We selected 13 NET-related genes to create a prognostic risk model after calculating with univariate, LASSO, and regression of multivariate logistic algorithms. Additionally, we investigated the immune cells based on the risk groupings and explored drug resistance patterns in patients with poor prognoses. The results suggested that dasatinib, a drug commonly used to treat relapsed leukemia, may still be effective for patients with poor prognosis in cALL. Furthermore, our findings illustrated potential roles for NET-related genes in contributing to the diminished effectiveness of cALL treatments. Based on these results, we further validated TRIM8, a gene selected to construct the prognostic model, in ALL cell line and ALL model of mice.

This is the first report investigating the role of TRIM8 in cALL and its relationship with NET-related genes. TRIM8 belongs to the tripartite motif protein family, characterized by the presence of three conserved domains, namely the coiled-coil region, RING domain, and B-box domain (26–28). TRIM8 has been implicated as an oncogenic protein, promoting cell proliferation through its interactions with NF-kB and STAT3 (29, 30). It facilitates the activation of NF-kB by promoting Lys63-linked polyubiquitination of TAK1 and subsequent IKK kinase activation (31). Moreover, TRIM8 is involved in the translocation of PIAS3 from the nucleus to the cytoplasm, therefore leading to its degradation. In the nucleus, PIAS3 connects with NF-kB, obstructing its activation. Furthermore, the degradation of two protein inhibitors of STAT, namely PIAS3 and SOCS-1, activates the JAK-STAT pathway via TRIM8 in response to IFN-γ stimulation. TRIM8 could enhance autophagy flux during the formation of lysosomes, leading to the suppression of cell death caused by genotoxic stress through the inactivation of the cleaved caspase-3 subunit. We then explored the expression of NF-kB and JAK-STAT in our groups using the TARGET database (**Supplementary Figure S1**). The results showed the NF-kB pathway was activated in the high-risk group, while the JAK-STAT pathway was at a low expression level in the high-risk group. Also, TRIM8 stabilizes the X-linked inhibitor of apoptosis (XIAP), a molecule modulating cell death and autophagy, thereby promoting the survival of cancer cells (32). These mechanisms collectively suggest TRIM8’s potent oncogenic potential and its ability to confer survival advantages to cancer cells (33).

NETs are considered to have critical roles in immune evasion, tumor encapsulation, and the protection of cancer cells from immune surveillance, such as NK cells and CD8+ T cells (9). NETs act as a protective shield that hinders the effectiveness of immune checkpoint inhibitors and CAR-T cell therapy (34). Furthermore, studies have reported that NETs can activate NF-kB, which is a common event in most hematologic malignancies (35, 36). Additionally, autophagy, a cellular process, can regulate several neutrophil functions, including differentiation, lifespan, apoptosis, degranulation, and NET formation (37, 38). The release of NETs can be modulated by autophagy. Considering that cell death is the beginning of releasing NETs, we assume that the death of neutrophils is the reason for downregulating TRIM8. A study has demonstrated NETs were increased after chemotherapy as the drugs used in treatments are lethal for neutrophils, leading to the release of NETs (39). Thus, the expression level of NETs determines the effect of chemotherapy, reflecting the prognosis of patients with ALL. The releasing NETs change many molecules, and we validated TRIM8 as an important factor influencing the development of ALL during the activation of NETs. The common path of cell death could be the potential mechanism between NET-related genes and TRIM8. Our study provides evidence supporting improved prognosis in cALL patients upon TRIM8 knockdown and highlights its potential as a predictive marker.

However, whether NET-related genes directly activate TRIM8 remains to be elucidated. Our analysis suggests a potential interaction between NET-related genes and TRIM8-associated pathways. Nevertheless, this represents a limitation of our study, and further experimental investigations are warranted to elucidate the specific genes in NET-related genes and pathways.

## 5 Conclusion

To summarize, this study investigated the role of NET-related genes in cALL and developed a prognostic model based on their signature. The findings suggest that the TRIM8 gene plays a significant role in the prognosis of cALL, and its knockdown improved the prognosis in ALL models, indicating its potential as a therapeutic target for cALL.

## Data availability statement

The datasets presented in this study can be found in online repositories. The names of the repository/repositories and accession number(s) can be found in the article/**Supplementary Material**.

## Ethics statement

The animal study was approved by Institutional Animal Care and Use Committee at Sun yat-sen University (agreement number: SYSU-IACUC-2024-B0726). The study was conducted in accordance with the local legislation and institutional requirements.

## Author contributions

WT: Formal analysis, Writing – original draft. CX: Conceptualization, Methodology, Writing – review & editing. KS: Methodology, Software, Writing – review & editing. YZ: Data curation, Visualization, Writing – review & editing. MX: Formal analysis, Writing – review & editing. JZ: Investigation, Writing – review & editing. YW: Resources, Writing – review & editing. SL: Funding acquisition, Project administration, Writing – review & editing. UY: Conceptualization, Funding acquisition, Writing – review & editing.
