# Supplementary material for: TRIM8 as a predictor for prognosis in childhood acute lymphoblastic leukemia based on a signature of neutrophil extracellular traps
Source: Front Oncol. 2024 Aug 19;14:1427776. doi: 10.3389/fonc.2024.1427776 (PMC11366590; doi:10.3389/fonc.2024.1427776)
Supplement: Supplementary file 1 [file Datasheet1.docx]

**Supplementary Material**

**Supplementary Figure S1**


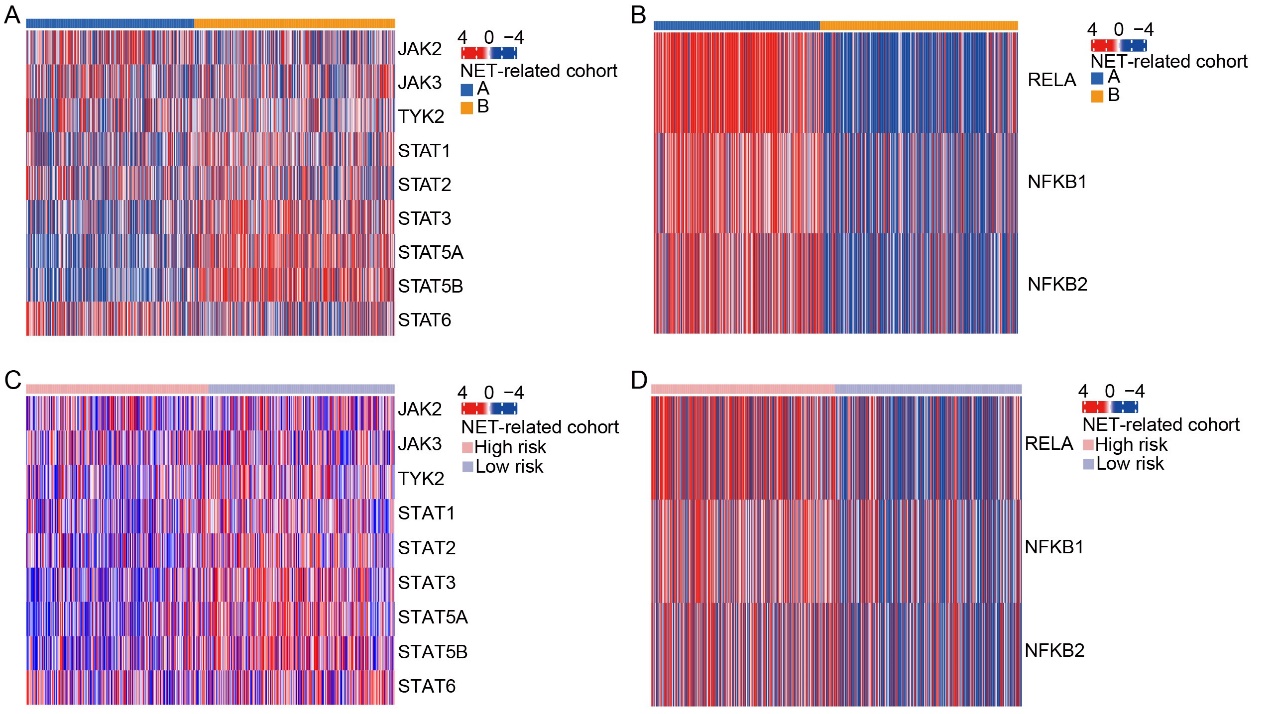


(A-B) Expression level of JAK.STAT and NF-KB between NET-related groups. (C-D) Expression level of JAK.STAT and NF-KB between high-risk group and low-risk group of NET-related genes.
