# Supplementary material for: TRIM8 as a predictor for prognosis in childhood acute lymphoblastic leukemia based on a signature of neutrophil extracellular traps
Source: Front Oncol. 2024 Aug 19;14:1427776. doi: 10.3389/fonc.2024.1427776 (PMC11366590; doi:10.3389/fonc.2024.1427776)
Supplement: Supplementary file 3 [file Table2.docx]

**Supplementary Material**

**Supplementary Table S1**

The clinical characteristics of both the training cohort and the test cohort.

| **Variables** | **Group** | **Training set**  **（285）** | **Testing set**  **（284）** |
| --- | --- | --- | --- |
| **Age** | **<=20** | 280 | 280 |
|  | **>20** | 5 | 4 |
| **Gender** | **Female** | 105 | 104 |
|  | **Male** | 180 | 180 |
| **Vital status** | **Alive** | 205 | 214 |
|  | **Dead** | 80 | 70 |
| **Survival time** |  | 2785.758 | 2816.021 |
| **Ethnicity** | **Hispanic or latino** | 50 | 43 |
|  | **Not hispanic or latino** | 166 | 170 |
|  | **Unknown** | 69 | 71 |
| **Race** | **Asian** | 15 | 4 |
|  | **Black or african american** | 25 | 22 |
|  | **Native hawaiian or other pacific islander** | 3 | 1 |
|  | **White** | 160 | 175 |
|  | **Unknown** | 82 | 82 |
|  |  |  |  |
